# Intelligent IoT (IIoT) Device to Identifying Suspected COVID-19 Infections Using Sensor Fusion Algorithm and Real-Time Mask Detection Based on the Enhanced MobileNetV2 Model

**DOI:** 10.3390/healthcare10030454

**Published:** 2022-02-28

**Authors:** Rupali Kiran Shinde, Md. Shahinur Alam, Seong Gyoon Park, Sang Myeong Park, Nam Kim

**Affiliations:** 1 Department of Information and Communication Engineering, Chungbuk National University, Cheongju 28644, Korea; rups@chungbuk.ac.kr (R.K.S.); shahinur@chungbuk.ac.kr (M.S.A.); smpark11@korea.kr (S.M.P.); 2 Department of Smart Information Technology Engineering, Kongju National University, Gongju 32588, Korea; psk@kongju.ac.kr

**Keywords:** COVID-19, enhanced MobileNetV2, IoT device sensor fusion, suspect detection and s tracking, face mask detection, remote monitoring

## Abstract

This paper employs a unique sensor fusion (SF) approach to detect a COVID-19 suspect and the enhanced MobileNetV2 model is used for face mask detection on an Internet-of-Things (IoT) platform. The SF algorithm avoids incorrect predictions of the suspect. Health data are continuously monitored and recorded on the ThingSpeak cloud server. When a COVID-19 suspect is detected, an emergency email is sent to healthcare personnel with the GPS position of the suspect. A lightweight and fast deep learning model is used to recognize appropriate mask positioning; this restricts virus transmission. When tested with the real-world masked face dataset (RMFD) dataset, the enhanced MobileNetV2 neural network is optimal for Raspberry Pi. Our IoT device and deep learning model are 98.50% (compared to commercial devices) and 99.26% accurate, respectively, and the time required for face mask evaluation is 31.1 milliseconds. The proposed device is useful for remote monitoring of covid patients. Thus, the method will find medical application in the detection of COVID-19-positive patients. The device is also wearable.

## 1. Introduction

In December 2019, a pneumonia-like disease began to spread worldwide, accompanied by fever and cold-like symptoms [1,2], caused by the COVID-19 (Coronavirus disease of 2019) virus [3,4]. The World Health Organization (WHO) declared COVID-19 a Public Health Emergency of International Concern on 30 January followed by declaration of pandemic on 11 March 2020. pandemic affects people’s mental and physical health. To date, 401 million COVID-19 cases have been detected, with 5.76 million deaths confirmed. The increasing number of COVID-19 cases and deaths have led to worldwide lockdowns, quarantines, and restrictions on human movements. Abdulkadir Atalan mentioned that lockdowns could suppress the spread of the virus. Reference [4] also mentioned the effects of lockdowns on psychology, the environment, and the economy. Various studies have shown the effects of lockdowns on economics, domestic abuse, mental health, and social health [5].

Even though many types of vaccines are in the market, but there are new virus strains coming due to mutations. Vaccinating the entire world population is an ideal way to stop pandemics, but many countries are poor, and their healthcare systems are not advanced enough to provide vaccine for all population. Moreover, H.C Hsu presented the effects of COVID-19 on healthcare workers; for example, nurses are overworking and are under pressure; thus, it will take a long time to reach an ideal situation [6]. In the era of globalization, it has been difficult to travel during pandemic conditions. At present, the omicron strain is a major concern worldwide and many countries have announced restrictions on gathering and traveling, causing harm to economies and social welfare. Early testing and tracing are used to control the number of cases and outbreaks.

Here, we present an Internet-of-Things (IoT)-based device for early detection of infected subjects and to control spread via face mask detection. IoT devices collect and share data (with minimal human interaction) using various transfer protocols [7]. IoT applications are used in healthcare and smart factories, homes, and education. A fitness band is an IoT-based wearable device that monitors user activities and health. Lockdowns create economic and mental health difficulties [8]. This paper presents a wearable device that detects suspected COVID-19-infected individuals.

Dong et al. [9] developed a wearable device for continuous blood pressure monitoring [10]; this device did not store health data for future analysis. Aadil et al. [11] described a wireless body area network (WBAN) that used the IoT for remote health monitoring. A ZigBee network was implemented by Li et al. [12] to connect devices to a base station. Fu et al. [13] utilized a wireless sensor network and a Wi-Fi transmission protocol to measure blood oxygen levels in athletes but this paper focuses only on one health parameter, which makes overall health-checking difficult. The literature indicates that Wi-Fi protocols are appropriate and cost-effective for wearable devices. Artificial intelligence (AI) has played an important role during the pandemic. AI algorithms have been used to identify COVID-19 infections using features extracted from electrocardiograms or chest x-rays. Machine-learning algorithms that rapidly analyzed blood samples were 90% accurate when used to estimate the survival of COVID-19-infected patients [14,15]. M. Phan et.al. proposed a patent to detect COVID-19 using breathing data trained on IoT devices, but the sample size of the data was small [16]. Several authors have used deep learning techniques for face mask detection. The databases includes Kaggle, the face mask label dataset (FMLD), the masked face analysis (MAFA) dataset, and the real-world masked face recognition dataset (RMFRD) [17,18]. The YOLOv2, YOLOv3, SSDMNV2, MobileNetV2, and ResNet50 deep learning models for face mask detection are over 95% accurate. Some models are compatible with IoT platforms; others require high-performance graphic processing units (GPUs) [19,20,21].

This paper presents a preventive approach to avoid virus outbreaks and control the pandemic. The major contribution of this work is the application of the sensor fusion method for covid detection automatically using artificial intelligence. The proposed device takes percussion to avoid false-positive alerts. False-positives will create trouble for the healthcare system instead of helping it. The enhanced MobileNetV2 model is the optimal solution for IoT platforms due to the small model size, higher accuracy, and lower detection time.

Here, artificial intelligence (AI) is used to aid healthcare systems. This work detects and traces infected persons in real-time; this limits viral spread and outbreak. Automatic and correct locations of masks detected that control spread. This method is preventative and rapid. This paper is divided into five sections. Section 2 focuses on the proposed method, Section 3 presents the experimental setup, the results and discussion are presented in Section 4, and the conclusion and future scope are presented in Section 5.

## 2. The Methodology

**Figure 1:**
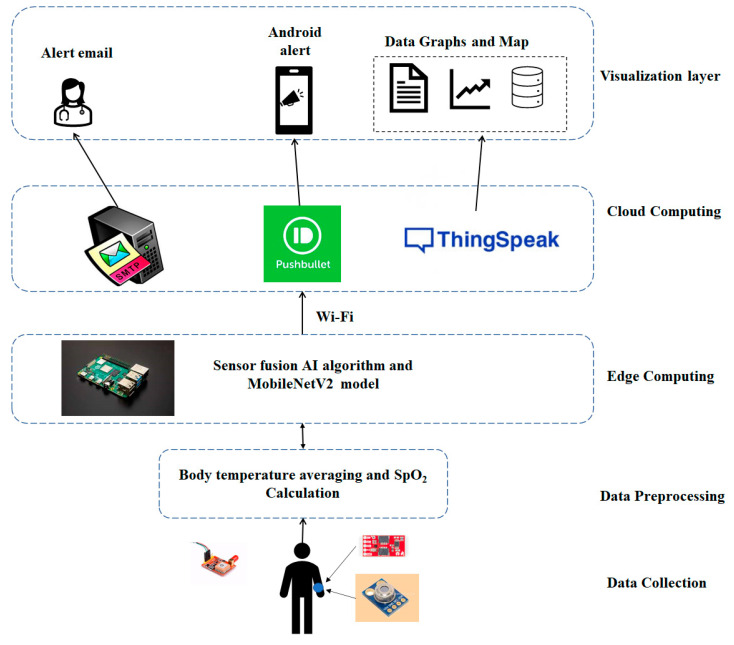
The overall architecture of the proposed IIOT device.

**Figure 2:**
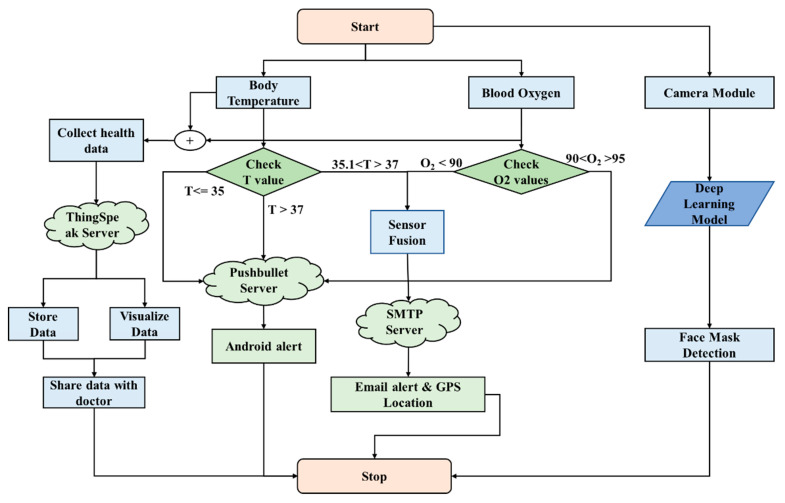
The data flow.

The proposed method uses a sensor fusion (SF) algorithm to detect infected suspects in the early stage of infection and detect face masks. We implemented a deep learning model on an IoT platform. The decision-making intelligence was provided by the SF algorithm and the deep learning model. Section 2.1 explains the SF algorithm and Section 2.2—face mask detection. The overall architecture of intelligent IoT (IIoT) devices is shown in Figure 1, with separate layers and the functionality of each layer. The data flow is shown in Figure 2, as well as the feature data collection and processing by the SF and deep neural network (DNN) algorithms, along with hardware and software components used in the system. SF merges sensory inputs from various channels to improve the information (compared to that available if the sources is used separately) [22]. SF finds applications in autonomous cars [23], robotics [24], and biomedical appliances [25]. To the best of our knowledge, this is the first work to use SF for COVID-19 disease prediction. The SF algorithm fuses inputs from blood oxygen, body temperature, and heart rate sensors. Low oxygen levels and fevers are the most common symptoms in COVID-19 patients; these are often misunderstood as normal colds in the early stages of the disease. Our method focuses on these three factors. Even if only one symptom is apparent, the AI algorithm sends an android alert of the unusual reading. The subject can now consider self-isolation and a possible need for medical care. The proposed approach does not detect asymptomatic people. This method does not confirm infection but, rather, anticipates who might be infected with COVID; this assists in early testing and tracing.

In this method, three different cloud servers are implemented for the respective functionality, as shown in Figure 1. ThingSpeak [26] is a cloud-based IoT platform that aggregates, visualizes, and analyzes real data streams. A private channel is created; the cloud provides a write API key used to save data, and a read API key to receive saved data in JSON, XML, or in text format. We installed the simple mail transfer protocol (SMTP) on the Raspberry Pi [27]. The SMTP server sends an alert email with crucial health data and the GPS position of a suspect to a healthcare provider. The Pushbullet server [28] is used to transfer links, text, and files between devices. This server sends android alerts that are not urgent but that require attention soon. After registering a device using its ID, the Pushbullet server delivers messages and notifications. Data collection and cloud storage are shown in Figure 2. The edge device features SF and notification servers. Real-time face detection (using a spy camera) predicts an output with the aid of the trained deep learning model (Figure 1).

### 2.1. Sensor Fusion (SF)

The sensor fusion (SF) approach is used to identify COVID-19 suspects. A body temperature of 35–37 °C is normal; an alarm is sent if the temperature exceeds this range. The normal blood oxygen level is 95–100%; anything below that range is considered serious. To generate emergency alerts, the data from the two sensors are fused and the threshold values evaluated. The SF algorithm and its implementation are shown in Algorithm 1.
**Algorithm 1.** Pseudo-code: COVID suspect prediction 1. Save the input from the temperature sensor; 2. If a finger is on the sensor, go to step 3; otherwise stop; 3. If sensor reading confidence level is above 90% collect data; 4. Save the input from the oximeter; 5. If 90 < O_2_ < 95: Send an android alert message via the Pushbullet server; 6. If O_2_ ≤ 90: If fever > 37.5: Assign Array [] and store the values for 30 min; If max of array [] < 90: Send an email stating that a suspect has been detected; include the GPS location; Otherwise, clear the array; else, send “low O_2_ need attention” alert to user; else, collect and save data in real-time.

SF algorithm features:

- The SF algorithm receives input data from fever, oximeter sensors, and heart rate, all of which are calibrated to commercial-level precision.
- To eliminate errors, the oximeter sensor accepts readings only when the sensor is in contact with human skin and the sensor’s confidence level is above 90%.
- When the oximeter indicates a low oxygen level, this might be transient (caused by exercise or stress). To avoid false positives, the SF system waits and examines additional health metrics.
- When the oxygen level drops, the system seeks information from the body temperature sensor.
- If both sensors produce anomalous results, the SF algorithm records all inputs for 30 min in an array and saves them for future study.
- If all values are below the usual levels for an extended period, only then does the SF algorithm send an email alert with a GPS position. If the values are not anomalous over an extended period, the algorithm concludes that no emergency exists, wipes all data from the array, and sends a simple notice to an Android smartphone.

### 2.2. Face Mask Detection Using Deep Learning on an IoT Platform

Deep learning is a form of image processing for AI that employs feature extraction algorithms. This requires a powerful GPU, but IoT devices lack a powerful GPU, which makes rendering deep learning difficult. Image processing employs the OpenCV and TensorFlow platforms. Raspberry Pi 4 includes support for image processing systems, such as Keras. MobileNetV2 [29] is an efficient neural network for IoT devices featuring an inverted residual structure with connections between the bottleneck levels, so we used this as a backbone network.

**Figure 3:**
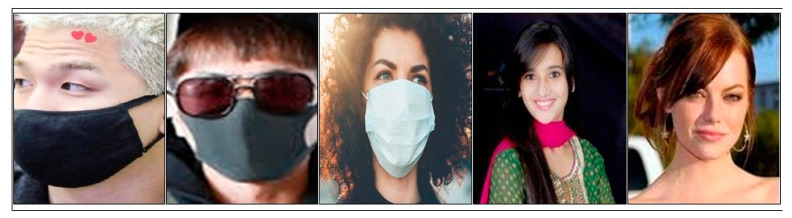
Sample images used for neural network training.

We used the RFMD dataset (which includes 2165 pictures with masks and 1930 without masks) for testing and training. Sample pictures are shown in Figure 3, along with pictures from the Bing search API and the Kaggle datasets. The manually morphed pictures are not included in the dataset; corrupt and duplicate pictures are removed. Cleaning, detection, and correction improved prediction. The dataset was divided into 80% for training and 20% for testing subsets before pre-processing. A function was implemented that accepted dataset folders as inputs, loaded all files, and resized the pictures. The list was then sorted alphabetically, and the pictures were transformed into tensors. The list was then transformed to a NumPy array (to accelerate computation).

The OpenCV library was used to recognize human faces rapidly before training. To eliminate recursive scan latency, several faces could be identified in a single shot; only one image was required to identify numerous objects. This determined the region of interest for MobileNetV2 feature extraction. Figure 3 presents sample images used to train the model. We had a diversified dataset with different nationalities, age groups, sexes, ethnicities, and types of masks for better accuracy.

**Figure 4:**
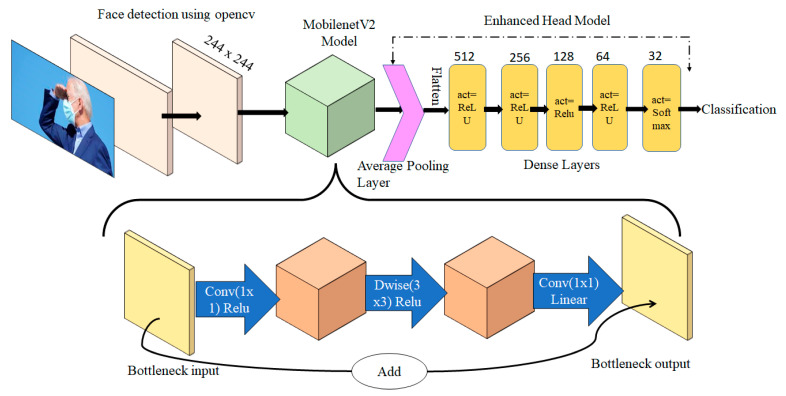
Face mask detection.

**Figure 5:**
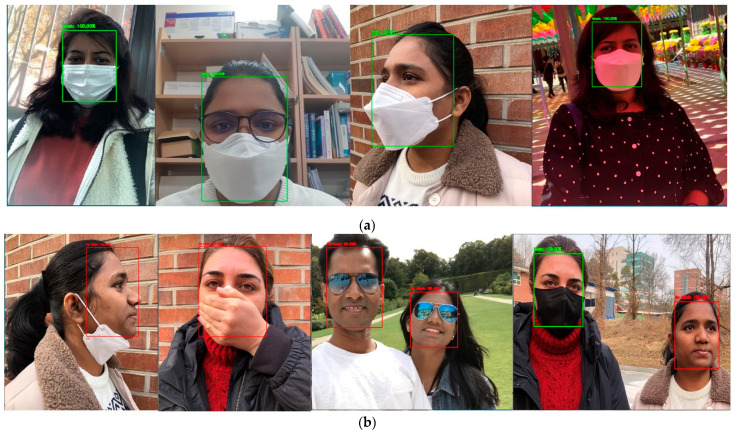
(**a**) Real-time face mask detection from different viewpoints. (**b**) Real-time face detection without a mask, capable of detecting the incorrect position of the mask and identifying it as “without mask”.

**Figure A1:**
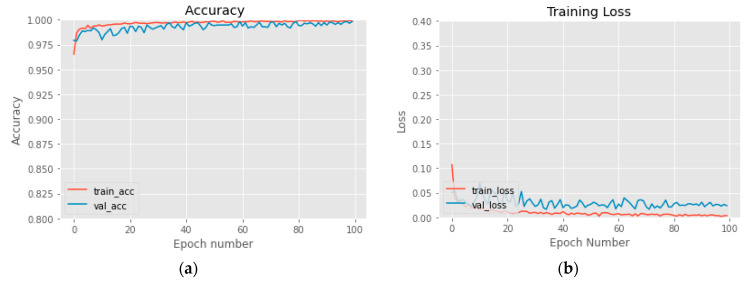
Accuracy (**a**) and loss (**b**) of the proposed model per epoch.

MobileNetV2 is a lightweight, deep learning neural network for picture classification. The standard MobileNetV2 model is in this work base model; the head model is added to enhance to base model output. The head model enhances the accuracy and it includes an averaging pooling layer followed by flattening operations. There were five dense layers added before the output layer. Whereas in the base model, TensorFlow was used to load the pre-trained weights. Then, to allow feature extraction, additional layers were added to (and trained on) the database. The model was then fine-tuned, and the weights were saved on the layers. Transfer learning saves time; existing biased weights were used without sacrificing previously learned features. MobileNetV2 features a core convolutional neural network layer. A pooling layer accelerates calculations by decreasing the size of the input matrix without changing its features. The dropout layer prevents overfitting during model training. The non-linear functions include several types of rectified linear units (ReLUs). The fully connected layers are linked to the activation layers. If connections are skipped, network execution may suffer. Thus, a linear bottleneck was added. Figure 4 shows the detailed architecture of the model. The method precisely identifies mask location. If a person is not wearing a mask, the model draws a red box around the face. The model can detect several faces in the same frame at the same time. This model can employ a basic picture as an input, or a real-time video stream from the Raspberry Pi camera. Figure 5 shows face mask detection and the percentage accuracies (red or green boxes). For critical analysis, images were taken from a side view and multiple faces on the same image to test the model. Figure A1a,b shows that face mask identification was 99.26% accurate; the loss and accuracy were plotted by the epoch, respectively. The Figure A1a,b shows that, after the 20th epoch, accuracy was close to 99.26%, and the “after loss” per epoch, was also minimum, which satisfied the well-fitted model condition. The time required to train the model on Raspberry Pi was almost twice that required when a PC equipped with a GeForce GTX 750 GPU, an Intel Core i5 processor, and 8 GB of RAM, were employed. After training, the real-time mask detection speeds on a PC and the IoT devices were identical. The model was tested by placing different objects on faces, altering the mask positions, and capturing faces from the side. Even in such unusual circumstances, model performance was unaffected.

## 3. Experimental

In a serial communication system, Raspberry Pi 4 plays the role of a host and an Arduino the role of a slave. The MLX 90614 sensor detects body temperature; the SparkFun sensor detects the blood oxygen level and heartbeat [30,31,32,33]. The GPS signal is detected by an LM80 sensor connected to a USB port. The MLX 90614 and SparkFun biosensors are integrated into the Raspberry Pi and the Arduino, respectively. The I2C protocol is used to link the biometric sensors. The spy camera is installed on the Raspberry Pi camera slot for real-time video-streaming and face mask recognition [34]. As we propose, this device for wearable purposes, a small size camera is necessary. The detailed pin connections with Raspberry Pi 4 and Arduino Uno are explained in Table A1 (Appendix A) and Table A2 (Appendix A) respectively.

**Figure 6:**
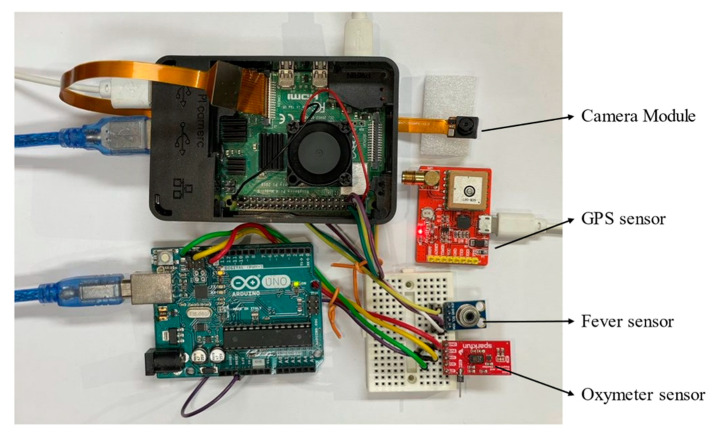
The experimental testbed.

Figure 6 shows the experimental setup. The Raspberry Pi 4 microprocessor is optimal for the TensorFlow platform. The analog sensor is powered by an Arduino Uno. To allow for future expansion, we used an Arduino rather than an analog-to-digital converter (ADC). During implementation, the multithreading feature of the Python language was used to effectively run the multiple sensors concurrently. There was a dedicated python thread; running concurrently for each sensor, Pi camera, and GUI data update featured.

Temperature sensor: the temperature sensor determines whether a person has a fever. Five hundred continuous inputs from the sensor are averaged in real-time before display to the user; the processing time is less than 1 s. A few milliseconds are required to provide the results, but health data are enormous; a short delay is acceptable. The enhancement algorithm is based on Equation (1):

(1) $$\mathrm{Output}\;\mathrm{temperature}=\sum_{1}^{n}\frac{\mathrm{Temp}}{n}$$

where temp = current temperature in Celsius and n = number of inputs.

The SparkFun sensor: the SparkFun sensor works as a pulse oximeter and the heart rate sensor is an I2C-based biometric sensor that features two Maxim Integrated chips; the MAX32664 sensor analyzes data collected by the MAX30101 sensor and the photoplethysmogram (PPG).

## 4. Results and Discussion

### 4.1. Device Performance

**Figure 7:**
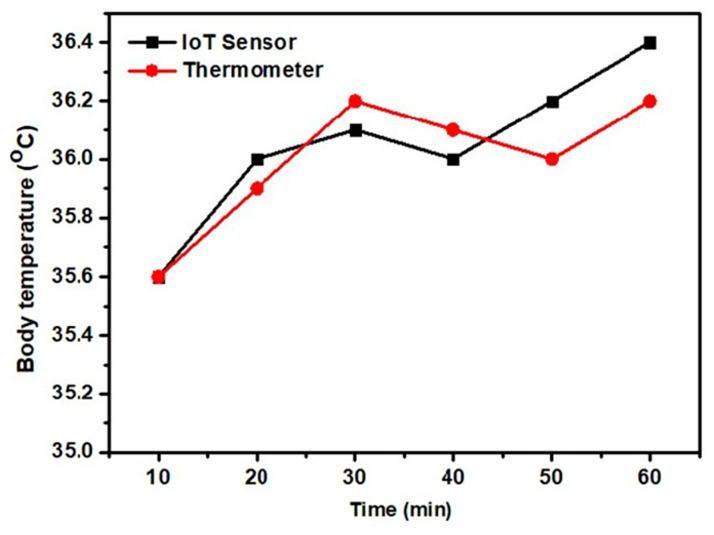
Comparison of the MLX 90614 sensor and the thermometer.

The accuracies of sensor data and face mask identification were evaluated. The MLX 90614 sensor was tested on the same individual; readings were obtained at 10-min intervals and compared to those of a commercial thermometer (Figure 7). All temperature measurements are in Celsius. The MLX 90610 sensor error was about 0.1 °C; the accuracy was thus about 98%. The temperature sensor gave the best accuracy when the user and sensor were stable.

**Figure 8:**
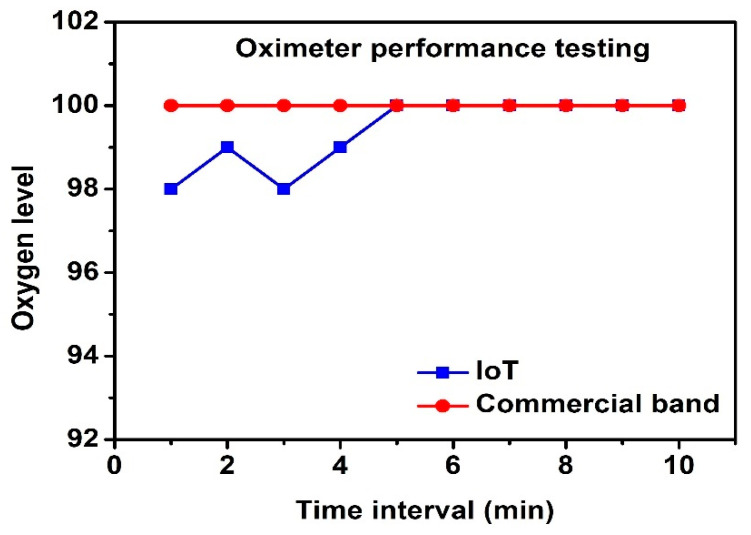
SparkFun sensor accuracy.

**Figure A2:**
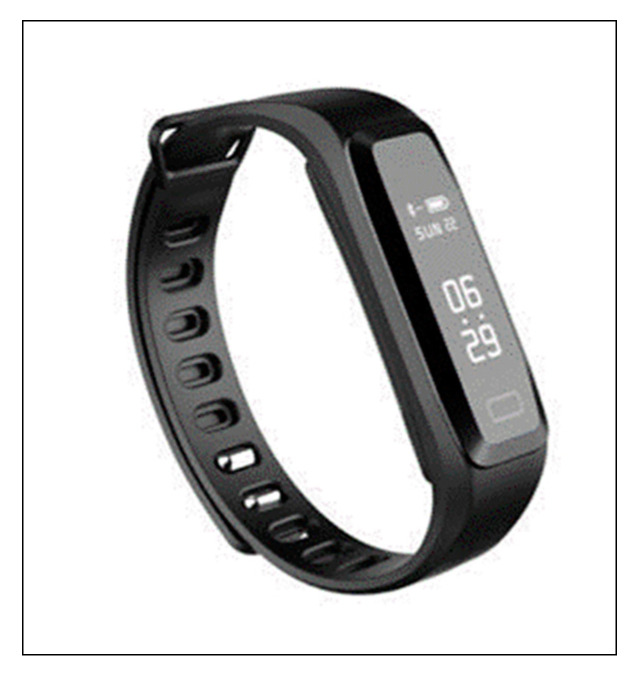
The Britz health band.

The SparkFun sensor is a pulse oximeter. The values obtained are plotted against those of the commercial Britz band (Figure 8). The picture of the commercial health band is shown in Figure A2 (Appendix A). The values were near-identical. The percentage accuracies at each time were averaged to yield an overall accuracy. Equation (2) shows the accuracy percentages at specific times; the average accuracy was then determined.

(2) $$\frac{\mathrm{IoT}\;\mathrm{value}\;}{\mathrm{Commercial}\;\mathrm{device}\;\mathrm{value}}\times \;100=\mathrm{Accuracy}\;\mathrm{percentage}$$

The average accuracy was 99.1%. The sensor also yielded the heart rate and raw data. Heart rate monitoring is critical in COVID-19-infected and cardiac patients because, according to Dr. Nisha Parekh, “There are numerous ways COVID-19 can damage the heart during the first period when someone has the infection, particularly in the first few weeks. These side effects might include new or worsening difficulties with blood pumping, inflammation of the heart muscle, and inflammation of the membrane around the heart. It should be emphasized that other infections can potentially cause the same symptoms.” [35]. Heart rate data were collected on the IoT server; however, the it was not included in suspected detection conditions.

**Figure A3:**
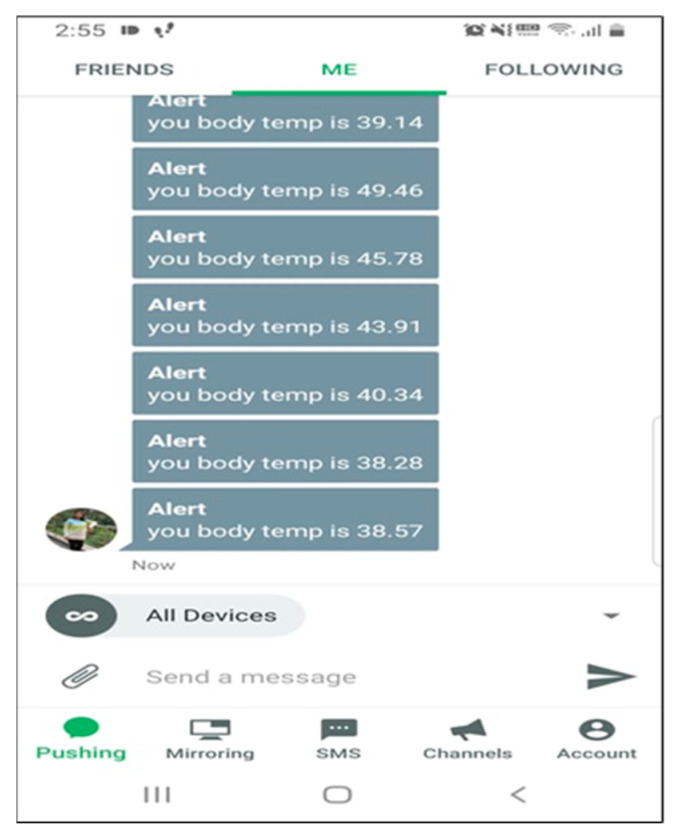
Android alert message.

**Figure A4:**
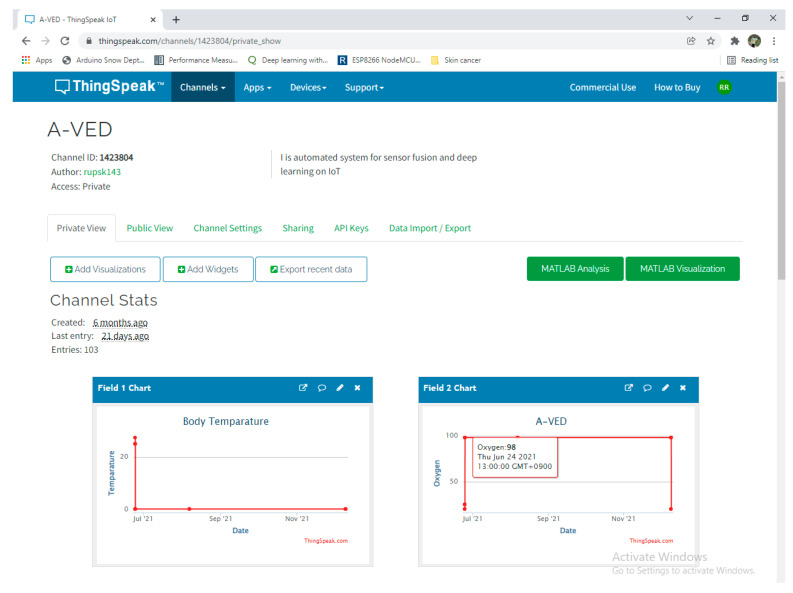
IoT cloud server ThingSpeak channel.

**Figure A5:**
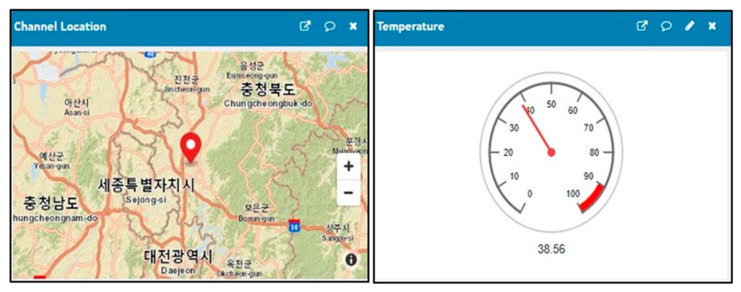
GPS and body temperature sensor data visualization on ThingSpeak cloud.

**Figure A6:**
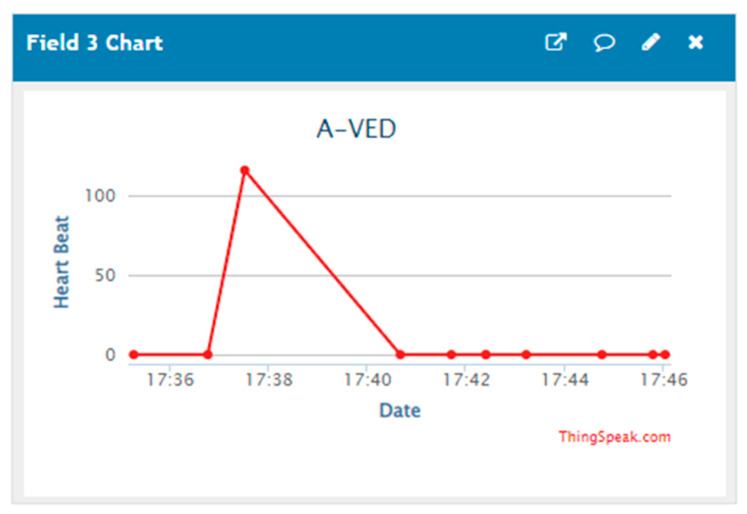
Heartbeat graph on IoT cloud.

An android message from the Pushbullet server is shown in Figure A3 (Appendix A). The android alert is issued only when the temperature falls below 30 °C or rises above 37 °C. Regarding the ThingSpeak channel connectivity and real-time data visualization is in MATLAB and each sensor value is represented as a single field and implementation output is provided in Figure A4 (Appendix A). The geographical position and the temperature are shown in Figure A5 (Appendix A). Heartbeat data were saved in field 3 of the ThingSpeak channel and values are plotted as shown in Figure A6 (Aappendix A). This shows our device is collecting data after every 15 min and saves over the cloud server. Along with data collection, data analysis is also performed over edge servers in real-time.

It is difficult to test the device on actual COVID patients due to social distancing rules; validation of the device was performed by Dr. Anuja Padwal, a practicing medical student at the Maharashtra University of Health Sciences (MUHS). According to Padwal, “The proposed method is beneficial for COVID perspective and automatic precautions for false positive is worth noting in the study. This method is beneficial and practical to control pandemics in developing countries because of the low manufacturing cost”.

The comparison of the our device with the available market devices are shown in Table 1, considering the various factors such as heart rate, body temperature, cost of the device, etc. 

### 4.2. Training and Testing of the Deep Learning Model

**Figure 9:**
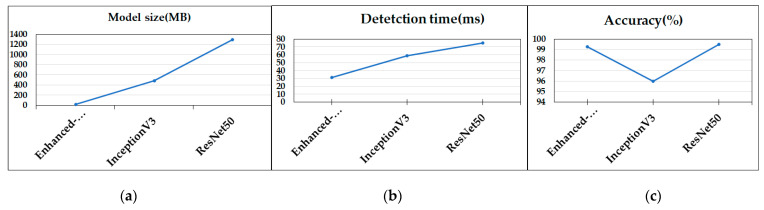
A comparison of the proposed model (enhanced MobileNetV2) with InceptionV3 and ResNet50 in terms of (**a**) size; (**b**) detection time; and (**c**) accuracy when evaluating the RMFD dataset.

For accuracy testing, we performed several tests of system performance, in terms of finding masked faces. For training purposes, the Adam optimizer with 30 epochs and a batch size of 32 was used. Loey et al. [26] evaluated training using Adam and SGDM and concluded that Adam outperformed SGDM in terms of a mini-batch root mean square error and loss. The Adam training is shown in Table 2; any loss was minor. Model performance was quantitatively compared to those of the InceptionV3 and ResNet50 architectures (using the RMFD dataset); the values are listed in Table 3 and plotted in Figure 9. The sizes of the deep learning model, the detection times, and the accuracies, were computed. Figure 9 shows that the ResNet50 architecture afforded the highest accuracy; however, this model includes more parameters than MobileNetV2, rendering it larger and slower. Figure 9c shows that the MobileNetV2 architecture is lightweight, with a size of 11.3 MB and a detection speed nearly half that of the ResNet50 model.

The training and validation loss curve is shown in Figure A1b. We observed that our model neither overfits nor underfits. Generally, the cost function is a way to compute error and to quantify how good or bad the model is performing. The less the loss, the more accurate the model is. From Figure A1b and Table 2, it could be concluded that the model is fine-tuned with minimal loss. In this experiment, the binary cross entropy function was used to optimize the model; the formula of the function is as given in Equation (3).

(3) $$\mathrm{Log}\;\mathrm{loss}=\frac{1}{N}\sum_{i=1}^{N}-\left(Yi*\log\left(pi\right)+\left(1+Yi\right)*\log(1-pi\right))$$

Here, *pi* is the probability of class with mask and (1 − *pi*) is the probability of class without a mask.

The model was further evaluated using the properly wearing masked face detection (PWMFD) dataset and compared with the results of Loey et al. [21]. Table 4 shows that the MobileNetV2 model size was the smallest and that our improvements reduced the detection time. The model accuracy using the RMFD dataset, PWMFD dataset, and combined dataset was only 99.11%, 89.00%, 90.14%, respectively, but when tested against the enhanced model, the accuracy was 99.26%, 99.15%, and 92.51%, respectively. We conclude that the enhanced model gives better accuracy with both datasets. The RMFD dataset performed better than PWMFD in all instances because many PWMFD pictures were blurred, rendering single-shot face identification difficult. Table 4 compares our system to that of Loey et al. [21].

In Table 4, we compare our model with other papers to show that the proposed model outperforms previously reported models. Whereas in Table 5, we combine RMFD and PWMFD datasets to compare the results of using the proposed model. In all instances, enhanced MobileNetV2 performs better than any other model. In [34], the authors presented face mask detection using SSD-MobileNetV2 and had 92.64% accuracy, whereas the presented model had 99.26% accuracy; hence, we can conclude that our model is accurate and lightweight compared to the other proposed models, which makes it suitable for IoT devices.

**Figure 10:**
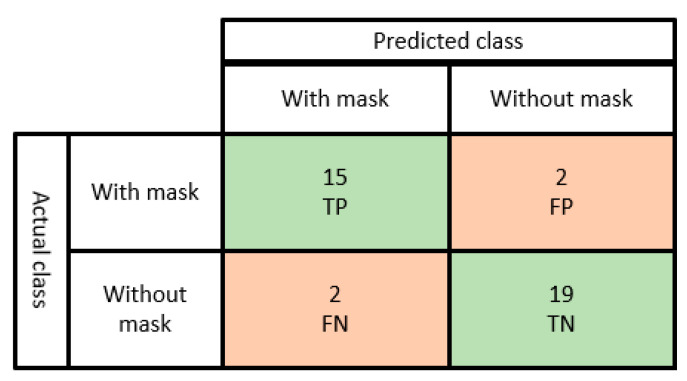
Confusion matrix for face mask detection model.

To further evaluate the model, we calculated true positive (TP), true negative (TN), false positive (FP), and false negative (FN) on 30 random images with 38 random faces. The confusion matrix is shown in Figure 10. The experiment results show that 15 TP, 19 TN, 2 FP, and 2 FN were detected. Additionally, the precision and recall were calculated based on Equations (4) and (5). The values of the precision and recall were 0.88 and 0.88, respectively.

(4) $$\mathrm{Precision}=\frac{\mathrm{TP}}{\mathrm{TP}+\mathrm{FP}}=0.88$$

(5) $$\mathrm{Recall}=\frac{\mathrm{TP}}{\mathrm{TP}+\mathrm{FN}}=0.88$$

Here, FP and FN values are low, meaning we could predict that the algorithm is precise and accurate, with a large-sized dataset; we are expecting higher TP and TN values.

## 5. Conclusions

We present a novel SF technique embedded in a device with a deep neural network; this method “seeks” ways to help control a pandemic. The accuracy of the device is 98–99%, compared to commercial devices. To avoid false-positive alerts, precautionary measures were automatically taken by the SF algorithm without human interference (key features of this paper). The proposed method identifies suspected COVID-infected individuals in real-time, and facilitates tracing and tracking using a GPS sensor. The presented method is economical, practical, scalable, easy to use, and pandemic-focused. To the best of our knowledge, this method is the first to implement SF technology in a wearable device for pandemic control. The proposed device mainly has application in two major categories—wearable gadgets and devices for public areas. Wearable devices can be used by COVID-19 patients or those with other critical conditions who require continuous real-time data monitoring in the absence of a doctor. If the device is used in public places (e.g., schools, malls, train and bus stations, airports, tourist places), face mask detection would ensure that people wear their masks correctly. The device is scalable, inexpensive, simple to deploy, user-friendly, and securely saves health data. Remote monitoring (without face-to-face medical consultation) is possible; continuously recorded data are shared. The read data API key allows a user to control the data completely; anyone else needs specific permission to view the data.

In the future, we will enhance device accuracy and attempt to reduce the size of the wearable device to make it more user-friendly. Furthermore, we plan to include additional sensors with microprocessors for other types of diseases, such as diabetics and cardiac arrest. IoT devices are vulnerable to cyber-attacks. Thus, data flowing from the device to the cloud must be encrypted and, therefore, security measures need to be added to prevent cyber-attacks. Health data are “big data”; data storage and access are challenging and researchers aim to address these issues.

## Figures and Tables

**Table 1:** Comparative study of the commercial device and proposed device.

Device Features	Apple Watch Series 6	Apple Watch Series 5	Proposed Device
Heart rate	✓	✓	✓
Body temperature	×	×	✓
Oximeter	✓	×	✓
Charging	Wireless	Wireless	USB
Database	Apple app	Apple app	IoT cloud
Data visualization	×	×	✓
Data sharing	×	×	✓
Alert and notification	×	×	✓
Sensor fusion for AI	×	×	✓
Covid suspect tracking	×	×	✓
Price (USD)	400+	400+	100

**Table 2:** Training and validation of the enhanced MobileNetV2 model on Adam.

Epoch	Iteration	Training Time (s)	Batch Loss	Accuracy (%)	F1 Score
5	120	569.19	0.0711	98.29	0.98
10	240	1164.06	0.0420	98.46	0.99
15	360	1709.57	0.0336	98.90	0.99
20	480	2165.29	0.0305	99.15	0.99
25	600	2538.23	0.029	99.20	0.99
30	720	3248.43	0.025	99.26	0.99

**Table 3:** Comparison of model sizes and detection times.

Model	Model Size (MB)	Detection Time (ms)	Accuracy (%)	Raspberry Pi Support
MobileNetV2	11.3	31.3	99.11	✓
InceptionV3	478.08	58.8	96.00	×
ResNet50	1296.62	74.9	99.51	✓
Enhanced MobileNetV2	11	31.3	99.26	✓

**Table 4:** Real-time face mask detection model comparative study.

Method	Backbone	Input Image Size	Detection Time (ms)	Accuracy %	Raspberry Pi Support
RetinaNet	ResNet-50	800	76.8	94.9	✓
EfficientDet-D0	EfficientDet-Bo	512	99.3	84.5	×
EfficientDet-D1	EfficientDet-B1	608	122.0	85.1	×
SSD	VGG-16	512	34.5	92.7	×
YOLOv3	Darknet53	608	61.5	95.3	✓
SE-YOLOv3	SE-Darknet53	512	49.2	96.2	×
MobileNet	MobileNetV2	512	31.9	90.1	✓
Enhanced-Mobile net	MobileNetV2	512	31.9	95.	✓

**Table 5:** Enhanced MobileNetV2 compared with MobileNetV2 against different datasets.

Model Name	Accuracy (%)		
	RMFD Dataset	PWMFD Dataset	RMFD + PEMFD Combine Dataset
MobileNetV2	99.11	89.00	90.14
Enhanced MobileNetV2	99.26	91.15	92.51

## Data Availability

The dataset and python code are available at https://github.com/shahinur-alam/Covid-Project. The ThingSpeak cloud channel is available at https://thingspeak.com/channels/1423804/private_show (Last accessed on 20 February 2022).

## Appendix A

Raspberry Pi 4 connections with sensors are connected via a I2C protocol using GPIO pins.

**Table A1:** Pin connections of Raspberry Pi 4.

Raspberry Pi 4	IoT Device
Body temperature sensor	
5V	VCC
Pin 6	GND
GPIO 2 Serial Data	SDA
GPIO 3 Serial Clock	SCL
LCD screen	
5V	VCC
Pin 7	GND
GPIO 17	SDA
GPIO 27	SCL
Alert buzzer	
GPIO	Positive
Pin 39	GND

Arduino Uno works as an analog sensor data collection and preprocessing analog data.

**Table A2:** Arduino Uno pin connection.

Arduino Uno	Oximeter Sensor
3.3 V	VCC
GND	GND
Analog (A4)	SDA
Analog (A5)	SCL

Human health-related vital data sometimes show abnormal readings, e.g., concerning abnormal condition emergency alerts, which are sent to users and relatives. These alerts will be useful for healthcare workers and in remote monitoring.

Fever and low oxygen are common signs of COVID-19; when both conditions occur at the same time, emergency tracing and testing is needed. To provide emergency services, location and data history are provided to healthcare workers through a read API key of the IoT cloud server.
